# Viral Lysis of Photosynthesizing Microbes As a Mechanism for Calcium Carbonate Nucleation in Seawater

**DOI:** 10.3389/fmicb.2016.01958

**Published:** 2016-12-09

**Authors:** John T. Lisle, Lisa L. Robbins

**Affiliations:** U. S. Geological Survey, St. Petersburg Coastal and Marine Science CenterSt. Petersburg, FL, USA

**Keywords:** homogeneous nucleation, viruses, photosynthetic microorganisms, calcium carbonate

## Abstract

Removal of carbon through the precipitation and burial of calcium carbonate in marine sediments constitutes over 70% of the total carbon on Earth and is partitioned between coastal and pelagic zones. The precipitation of authigenic calcium carbonate in seawater, however, has been hotly debated because despite being in a supersaturated state, there is an absence of persistent precipitation. One of the explanations for this paradox is the geochemical conditions in seawater cannot overcome the activation energy barrier for the first step in any precipitation reaction; nucleation. Here we show that virally induced rupturing of photosynthetic cyanobacterial cells releases cytoplasmic-associated bicarbonate at concentrations ~23-fold greater than in the surrounding seawater, thereby shifting the carbonate chemistry toward the homogenous nucleation of one or more of the calcium carbonate polymorphs. Using geochemical reaction energetics, we show the saturation states (Ω) in typical seawater for calcite (Ω = 4.3), aragonite (Ω = 3.1), and vaterite (Ω = 1.2) are significantly elevated following the release and diffusion of the cytoplasmic bicarbonate (Ω_calcite_ = 95.7; Ω_aragonite_ = 68.5; Ω_vaterite_ = 25.9). These increases in Ω significantly reduce the activation energy for nuclei formation thresholds for all three polymorphs, but only vaterite nucleation is energetically favored. In the post-lysis seawater, vaterite's nuclei formation activation energy is significantly reduced from 1.85 × 10^−17^ J to 3.85 × 10^−20^ J, which increases the nuclei formation rate from highly improbable (<<1.0 nuclei cm^−3^ s^−1^) to instantaneous (8.60 × 10^25^ nuclei cm^−3^ s^−1^). The proposed model for homogenous nucleation of calcium carbonate in seawater describes a mechanism through which the initial step in the production of carbonate sediments may proceed. It also presents an additional role of photosynthesizing microbes and their viruses in marine carbon cycles and reveals these microorganisms are a collective repository for concentrated and reactive dissolved inorganic carbon (DIC) that is currently not accounted for in global carbon budgets and carbonate sediment diagenesis models.

## Introduction

The increasing trend in studies of atmospheric *p*CO_2_ and ocean acidification (Doney et al., 2009) has focused efforts to characterize the interactions between shallow marine carbonate sediment systems and the overlying water and how these interactions alter DIC budgets (Krumins et al., 2013). Carbonate chemistry is most often the focus of these studies as calcium carbonate (Equation 1) is critical to the development and structural integrity of corals, shells, calcified algae, and sponges and directly influences global dissolved inorganic carbon (DIC) budgets (Feely et al., 2004).

(1) $$\begin{array}{l} \text{C}{{\text{a}}^{2+}}_{\left(\text{aq}\right)}+2\text{HC}{{\text{O}}^{-}}_{3\left(\text{aq}\right)}↔\text{CaC}{\text{O}}_{3\left(\text{s}\right)}+\text{C}{\text{O}}_{2\left(\text{aq}\right)}+\;{\text{H}}_{2}\text{O} \end{array}$$

The most common descriptor of calcium carbonate concentrations in marine water in the context of ocean acidification is the saturation state (Ω) (Equation 2),

(2) $$\begin{array}{l} \Omega ={\left[\text{C}{\text{a}}^{2+}\right]}_{\text{sw}}{\left[\text{C}{{\text{O}}_{3}}^{2-}\right]}_{\text{sw}}/{\text{K}}_{\text{sp}}^{*} \end{array}$$

where [Ca^2+^] and [${\text{CO}}_{3}^{2-}$] are the activity products of the calcium and carbonate ions, respectively, and ${\text{K}}_{\text{sp}}^{*}$ is the solubility product for calcium carbonate. Calcium carbonate has several polymorphs, each having a unique ${\text{K}}_{\text{sp}}^{*}$. The polymorphs and their respective ${\text{logK}}_{\text{sp}}^{*}$ at 25°C and listed in decreasing order of solubility in seawater include: amorphous calcium carbonate (ACC; ${\text{logK}}_{\text{sp}}^{*}$ = −6.280; Brecevic and Nielsen, 1989), vaterite (${\text{logK}}_{\text{sp}}^{*}$ = −7.913; Plummer and Busenberg, 1982), aragonite (${\text{logK}}_{\text{sp}}^{*}$ = −8.336; Plummer and Busenberg, 1982), and calcite (${\text{logK}}_{\text{sp}}^{*}$ = −8.480; Plummer and Busenberg, 1982).

Typical surface seawater Ω values range from 2 to 4, which is considered supersaturated. Interestingly, persistent precipitation events do not occur (Morse et al., 2003). This metastability of calcium carbonate in supersaturated seawater is due to geochemical conditions being unfavorable for overcoming the energetic threshold for the direct precipitation from seawater (i.e., homogeneous precipitation) or to a lesser extent on pre-existing surfaces (i.e., heterogeneous precipitation; De Yoreo and Vekilov, 2003; Radha and Navrotsky, 2013).

Existing models for the precipitation of calcium carbonate in seawater would be categorized as heterogeneous, where the existing surfaces include carbonate sediment particles (Broecker et al., 2000) and the outer membranes (e.g., S-layers) and extracellular polymeric substances (EPS) of microorganisms (Schultze-Lam et al., 1992; Thompson et al., 1997; Hodell et al., 1998; Thompson, 2000; Konhauser and Riding, 2012). Models for the homogeneous precipitation of calcium carbonate have also been proposed, in which the microbial processes of sulfate reduction, ammonification, and photosynthesis have been implicated (Cloud, 1962; Shinn et al., 1989; Glenn et al., 1995). The consensus explanation was though these processes were consistently measurable, they were not adequately proficient at altering native seawater geochemistry (e.g., pH, DIC) beyond the immediate surface of the active cells to the point where homogeneous precipitation events would proceed.

The cited studies and models describe different mechanisms for carbonate crystal precipitation and growth. However, they do not characterize the initial step that must occur in heterogeneous and homogeneous precipitation; nucleation. We propose there is a microbially induced pathway for calcium carbonate nucleation in seawater that is currently undetected due to extremely fast reaction times and small volumes. The proposed model compliments existing heterogeneous precipitation models while providing a novel mechanism through which homogeneous nucleation occurs. This model does not replace existing carbonate precipitation models, but provides an additional pathway through which solid phases of this mineral can be produced. Additionally, there was no attempt to quantify or include heterogeneous nucleation which initiates at much lower activation energies than those presented in this model (De Yoreo and Vekilov, 2003; Radha and Navrotsky, 2013). Our model is based on the naturally occurring interaction between photosynthetic microorganisms and their respective viruses which results in the lysis of the host microorganism and release of its intracellular contents, including bicarbonate (${\text{HCO}}_{3}^{-}$; Danovaro et al., 2011). The concentration of the intracellular ${\text{HCO}}_{3}^{-}$ is significantly greater than that in the surrounding seawater and upon release shifts the carbonate chemistry to the point of overcoming energetic thresholds, favoring nucleation of one or more of the calcium carbonate polymorphs.

The majority of photosynthesizing prokaryotic and eukaryotic microorganisms utilizes membrane associated carbon concentrating mechanisms to preferentially elevate concentrations of DIC (i.e., ${\text{HCO}}_{3}^{-}$) in the cytoplasm. Using cyanobacteria as an example, the cytoplasmic ${\text{HCO}}_{3}^{-}$ diffuses into carboxysomes where it is instantaneously converted to CO_2_ by carbonic anhydrase. This CO_2_ is then available to ribulose-1, 5-bisphosphate carboxylase/oxygenase (RuBisCO), the enzyme central to carbon fixation and photosynthesis (Price, 2011; Mangan and Brenner, 2014). The cytoplasmic ${\text{HCO}}_{3}^{-}$ concentration in photosynthesizing cyanobacteria has been measured at 40 mM (range: 30–50 mM; Price et al., 2008; Konhauser and Riding, 2012), while concentrations of Ca^2+^ and protons are kept at non-detectable levels by being actively exported. These membrane associated processes collectively prevent precipitation of calcium carbonate, while maintaining an average pH of 8.2 (range: 8.0–8.3) in the cytoplasm (Price et al., 2008; Konhauser and Riding, 2012).

Though there are several groups of photosynthesizing prokaryotes and eukaryotes in marine waters, we chose *Procholorcoccus* sp. and *Synechococcus* sp. as representative microorganisms because these are the two most numerically dominant photosynthesizing cyanobacteria in seawater and their interactions with marine viruses are well-characterized (Suttle and Chan, 1994; Danovaro et al., 2011). These genera have an average total cell volume of 0.191 μm^3^, which is 40% (v/v) aqueous. The cytoplasmic ${\text{HCO}}_{3}^{-}$ is dissolved within this volume (7.60 × 10^−14^ cm^3^; Scanlan, 2012). Upon infection of the host cells by their virulent or temperate viruses, a number of cytopathic effects quickly occur following the initiation of the lytic cycle, though this initiation can be delayed in lysogenic populations. Regardless of the timing of the initiation, the lytic cycle culminates in the instantaneous and violent “bursting” of the host cell's outer membrane (Young, 2014). The ruptured cells not only release progeny viruses but also cytoplasmic ${\text{HCO}}_{3}^{-}$ at a concentration that can be ~23-fold greater than that in the surrounding seawater (1.77 mM; Zeebe and Wolf-Gladrow, 2013).

The reaction between the released ${\text{HCO}}_{3}^{-}$ and Ca^2+^ from the surrounding seawater is the first step in the nucleation process. This reaction was modeled as a diffusion controlled reaction, where the Ca^2+^ concentration (10.28 mM) in the surrounding seawater (Zeebe and Wolf-Gladrow, 2013) is isotropic and infinite. The second order reaction rate constant (*k*_*D*_; M^−1^s^−1^) for the CaCO_3_ molecule formation reaction was derived using the following relationship (Equation 3) (Eigen and Hammes, 1963; Caldin, 2001):

(3) $$\begin{array}{l} k_{D}\;=\;\frac{4\pi N_{A}a\;(D_{A}+D_{B})\;(z_{A}z_{B}e_{0}^{2}/\varepsilon_{0}\varepsilon ak_{B}T)}{\text{exp }\left(z_{A}z_{B}e_{0}^{2}/\varepsilon_{0}\varepsilon ak_{B}T\right)-1} \end{array}$$

where *N*_*A*_ is Avagadro's constant (6.02 × 10^23^ molecules mole^−1^); *a* is the distance of closest approach between two ions, which in this case is the sum of the ionic radii for ${\text{HCO}}_{3}^{-}$ (1.56 × 10^−8^ cm) and Ca^2+^ (1.00 × 10^−8^ cm); *D*_*A*_ and *D*_*B*_ are the diffusion coefficients for ${\text{HCO}}_{3}^{-}$ (1.18 × 10^−5^ cm^2^ s^−1^) and Ca^2+^ (7.93 × 10^−6^ cm^2^ s^−1^), respectively (Li and Gregory, 1974); *z*_*A*_ and *z*_*B*_ are the ionic charges of ${\text{HCO}}_{3}^{-}$ (−1) and Ca^2+^ (+2), respectively; *e*_0_ is the elementary charge of an electron (1.60 × 10^−19^ coulombs); ε_0_ is the vacuum dielectric constant (8.85 × 10^−12^ farads m^−1^); ε is the dielectric constant for water (78.54 unit less); *k*_*B*_ is Boltzmann's constant (1.3805 × 10^−23^ J °K^−1^) and *T* is temperature (°K). The reaction rate constant for this reaction, *k*_*D*_ = 2.68 × 10^11^ M^−1^s^−1^, is similar in magnitude to those from other diffusion controlled reactions (Eigen and Hammes, 1963; Caldin, 2001). This rate constant is considered an upper limit as each collision between a ${\text{HCO}}_{3}^{-}$ and Ca^2+^ molecule is considered to be 100% efficient in producing a single CaCO_3_ molecule, thereby making the collision rate equal to the reaction rate.

Using this reaction rate constant in the following integrated rate equation for unequal concentrations of reactants (Equation 4):

(4) $$\begin{array}{l} \frac{1}{{\left[HCO_{3}^{-}\right]}_{0}-{\left[Ca^{2+}\right]}_{0}}ln\frac{{\left[Ca^{2+}\right]}_{0}}{{\left[HCO_{3}^{-}\right]}_{0}}\frac{{\left[HCO_{3}^{-}\right]}_{t}}{{\left[Ca^{2+}\right]}_{t}}=k_{D}t \end{array}$$

where [${\text{HCO}}_{3}^{-}$]_0_ = 40.0 mM, [${\text{HCO}}_{3}^{-}$]_*t*_ = 1.77 mM, [Ca^2+^]_0_ = 10.28 mM and [Ca^2+^]_*t*_ = 10.28 mM, the minimal time for this association reaction to proceed to the point where the ${\text{HCO}}_{3}^{-}$ released from the cell is in equilibrium with that in the surrounding seawater (1.77 mM) is 3.88 × 10^−10^ s.

The final volume in which this reaction reaches equilibrium is estimated by first determining the distance (*D*_*d*_, cm) each ${\text{HCO}}_{3}^{-}$ molecule diffuses during the reaction time (*t*), using the diffusion coefficient (*D*_*A*_) for ${\text{HCO}}_{3}^{-}$ (Equation 5),

(5) $$\begin{array}{l} D_{d}=\sqrt{6D_{A}t} \end{array}$$

This distance (1.66 × 10^−7^ cm) is added to the radius (2.63 × 10^−5^ cm) of the initial volume of ${\text{HCO}}_{3}^{-}$ (7.60 × 10^−14^ cm^3^) and that total radius is used to calculate the final reaction volume; 7.80 × 10^−14^ cm^3^. This volume contains the original concentration 40 mM ${\text{HCO}}_{3}^{-}$ or 2.41 × 10^−22^ molecules of ${\text{HCO}}_{3}^{-}$. Assuming all ${\text{HCO}}_{3}^{-}$ molecules reacted with a molecule of Ca^2+^ in this final reaction volume during the 3.88 × 10^−10^ s, a maximum number of 2.41 × 10^22^ molecules of CaCO_3_ would be formed.

In the first part of the model we described the formation rate for CaCO_3_ molecules within a specific volume of seawater. The second part of the model determines if the geochemical conditions within this volume are conducive to overcoming the activation energy barriers for the homogeneous nucleation of one or more of the CaCO_3_ polymorphs. We followed the classic nucleation theory and Ostwald's Rule of Stages, where the polymorph with the lowest activation energy will nucleate first, followed by the polymorph with the next lowest activation energy until equilibrium has been established (Stranski and Totomanow, 1933; Baumgartner et al., 2013). The variables which have the greatest influence on the activation energy barrier to nucleation are the saturation state (Ω) and interfacial surface tension (γ) (De Yoreo and Vekilov, 2003; Radha and Navrotsky, 2013). Though there is a general consensus on the appropriate K_sp_^*^ values for the different polymorphs of CaCO_3_, this is not the case for interfacial surface tension values, which range from 35 to 200 J cm^−2^ (Myasnikov et al., 2013). We chose an interfacial surface tension value for each of the polymorphs based on current literature in which characterizing homogenous nucleation events was the study's objective.

The activation energies (ΔG_a_; Joules) were calculated for homogeneous nucleation of calcite, aragonite and vaterite in the previously derived reaction volume (i.e., 7.80 × 10^−14^ cm^3^). The respective activation energies were then used to estimate the number of nuclei formed (J_N_; nuclei cm^−3^ s^−1^) within the same volume of seawater. A standard seawater composition (Zeebe and Wolf-Gladrow, 2013), with a salinity of 35 ppt, pH of 8.2 and 3.0 mg kg-SW^−1^ dissolved oxygen at 25.0°C, was used as a typical seawater at standard temperature and pressure. The post-lysis seawater composition was identical to that for typical seawater except for the addition of the intracellular 40 mM ${\text{HCO}}_{3}^{-}$ at a cytoplasmic pH of 8.2, which are common values for cyanobacteria (Price et al., 2008). The carbonate chemistry species and calcium activity products and corresponding Ω values were calculated using the React module within Geochemist's Workbench and the thermos_minteq.dat database (rel. 7.0.6).

Using these typical and post-lysis seawater geochemical conditions, the activation energies (ΔG_a_: Jouels) (Equation 6) and nuclei formation rates (J_N_: nuclei cm^−3^ s^−1^) (Equation 7) for homogenous nucleation of the calcium carbonate polymorphs were calculated (Stumm and Morgan, 1981; De Yoreo and Vekilov, 2003),

(6) $$\begin{array}{l} \Delta {\text{G}}_{\text{a}}=16\pi \gamma^{3}{\text{V}}^{2}/3{\left[{\text{k}}_{\text{B}}\text{T ln}\left(\text{Q}/\text{K}\right)\right]}^{2} \end{array}$$

(7) $$\begin{array}{l} {\text{J}}_{\text{N}}=\text{A exp }\left(-\Delta {\text{G}}_{\text{a}}/{\text{k}}_{\text{B}}\text{T}\right) \end{array}$$

where γ is the interfacial surface tension (calcite: 1.24 × 10^−5^ J cm^−2^; aragonite: 1.45 × 10^−5^ J cm^−2^; vaterite: 7.25 × 10^−6^ J cm^−2^; Söhnel and Mullin, 1978; Kralj et al., 1990; Manoli and Dalas, 2000), *T* is temperature (°K), k_B_ is Boltzmann's constant (1.3805 × 10^−23^ J °K^−1^), *A* is molecular collision frequency efficiency (10^30^ cm^−3^ s^−1^; Stumm and Morgan, 1981; De Yoreo and Vekilov, 2003) and *V* is the calculated molecular volume (calcite: 6.14 × 10^−23^ cm^3^; aragonite: 5.67 × 10^−23^ cm^3^; vaterite: 3.29 × 10^−23^ cm^3^).

The minimum number of CaCO_3_ molecules required to form critical sized nuclei were derived from the critical radius (r_c_) (Stumm and Morgan, 1981; De Yoreo and Vekilov, 2003) as follows (Equation 8),

(8) $$\begin{array}{l} {\text{r}}_{\text{c}}=2\gamma \text{V}/\left[{\text{k}}_{\text{B}}\text{T ln}\left(\text{Q}/\text{K}\right)\right] \end{array}$$

The critical radii were used to calculate the critical nuclei volumes (cm^3^). These volumes were then divided by the molecular volume (*V*) for the respective CaCO_3_ polymorphs, giving the number of CaCO_3_ molecules required to fill the critical nucleus volume.

The typical seawater conditions prior to the release of cytoplasmic ${\text{HCO}}_{3}^{-}$, do not favor the nucleation of any of the polymorphs, as their nucleation formation rates were all significantly <1.0 nuclei cm^−3^ s^−1^ and the nucleation times were significantly > 1.0 h (Table 1). The post-lysis conditions significantly increased the Ω values for calcite (Ω = 95.7), aragonite (Ω = 68.5), and vaterite (Ω = 25.9; Table 1) and decreased the activation energy thresholds for nuclei formation by ~10-, 14-, and 480-fold, respectively (Table 1). However, only vaterite nucleation is energetically favorable at a nucleation rate of 8.60 × 10^25^ nuclei cm^−3^ s^−1^ and taking ~1.7 × 10^−9^ s to form a nuclei composed of a critical number (*n* = 6) of CaCO_3_ molecules (Table 1).

**Table 1:** **Data on the homogeneous nucleation of CaCO_**3**_ in seawater from different sources**.

**Seawater source**	**Reaction rate constant (*k_*D*_*)**	**Reaction time**	**Reaction conditions**	**Form of CaCO_3_**	**Ω**	**Activation energy for nuclei formation (ΔG_a_)**	**Nuclei formation rate (J_N_)**	**Critical number of CaCO_3_ molecules**
	**M^−1^ s^−1^**	**s**				**Jouels**	**Nuclei cm^−3^ s^−1^**	**Molecules nuclei^−1^**
Mean composition seawater^1^	2.68 × 10^11^	3.88 × 10^−10^	Native	Calcite	4.3	3.37 × 10^−18^	5.81 × 10^−326^	1129
				Aragonite	3.1	7.75 × 10^−18^	<5.81 × 10^−326^	3367
				Vaterite	1.2	1.85 × 10^−17^	<5.81 × 10^−326^	60631
			Post-lysis	Calcite	95.7	3.42 × 10^−19^	8.82 × 10^−7^	36
				Aragonite	68.5	5.42 × 10^−19^	5.76 × 10^−28^	662
				Vaterite	25.9	3.85 × 10^−20^	8.60 × 10^25^	6
Bahama bank^2^	2.64 × 10^11^	1.55 × 10^−10^	Native	Calcite	3.7	4.10 × 10^−18^	9.90 × 10^−398^	1515
				Aragonite	2.7	9.96 × 10^−18^	<9.90 × 10^−398^	4904
				Vaterite	1.0	1.01 × 10^−15^	<9.90 × 10^−398^	2.46 × 10^7^
			Post-lysis	Calcite	90.7	3.41 × 10^−19^	2.63 × 10^−6^	36
				Aragonite	65.5	5.41 × 10^−19^	4.45 × 10^−27^	62
				Vaterite	25.2	3.83 × 10^−20^	1.03 × 10^26^	6
Makarov basin^3^	2.94 × 10^11^	1.04 × 10^−10^	Native	Calcite	0.7	NA^4^	NA	NA
				Aragonite	0.5	NA	NA	NA
				Vaterite	0.2	NA	NA	NA
			Post-lysis	Calcite	15.0	1.17 × 10^−18^	8.19 × 10^−106^	230
				Aragonite	10.3	2.14 × 10^−18^	5.88 × 10^−219^	490
				Vaterite	3.5	3.19 × 10^−19^	1.07 × 10^−7^	137

1 *Zeebe and Wolf-Gladrow (2013)*.

2 *Bustos-Serrano et al. (2009)*.

3 *Robbins et al. (2013)*.

4 *NA: not applicable due to the sea water at this site being undersaturated*.

Previous studies have documented the precipitation of calcium carbonate in seawater (Morse and He, 1993) or specifically aragonite (Sun et al., 2015) under laboratory conditions at a Ω = 18, which is similar to our modeled post-lysis Ω for vaterite (Table 1). However, Morse and He (1993) concluded their laboratory-based conditions for elevated saturation states could not develop or persist in natural seawater and therefore homogeneous nucleation in seawater was improbable. Our model demonstrates there is a mechanism through which the appropriate geochemical conditions for homogeneous nucleation and precipitation of calcium carbonate in seawater exists, though at reaction times and in volumes that were not previously considered.

Another argument against homogeneous nucleation of CaCO_3_ in seawater is the absence of detectable reductions in pH and alkalinity during naturally occurring calcium carbonate precipitation events (Morse et al., 1984, 2003). Based on the geochemical modeling of seawater conditions prior to and post-lysis, the pH in typical seawater did decrease from 8.20 to 6.35. However, the carbonate alkalinity remained elevated in the post-lysis seawater (835.5 mg kg·SW^−1^) relative to that in the seawater prior to cell lysis (82.2 kg·SW^−1^). We propose the residual alkalinity buffers the lowered pH to native levels within the reaction volume but has a negligible effect on pH or alkalinity outside the reaction volume. This buffering reaction would also be diffusion controlled with a reaction rate constant of ~10^11^ M^−1^ s^−1^ and an overall reaction time of ~10^−10^ s. We propose these changes in carbonate chemistry have gone undetected in the cited and similar studies because the methods used for quantifying pH and alkalinity are incapable of measuring these variables at the spatial and time scales of our model.

To test the applicability of the model, we analyzed two seawater sources that represent saturation state end members; Bahama Bank seawater (temperature = 28.7°C; pH = 8.13; salinity = 37 ppt; native [${\text{HCO}}_{3}^{-}$] = 1.59 × 10^−3^ M), in which spontaneous calcium carbonate precipitation events commonly occur (Bustos-Serrano et al., 2009); and the Makarov Basin (temperature = −1.58°C; pH = 7.73; salinity = 29 ppt; native [${\text{HCO}}_{3}^{-}$] = 1.85 × 10^−3^M), a region of the Arctic Ocean where ocean acidification has been documented and carbonate precipitation is unlikely (Robbins et al., 2013).

The calculated second order reaction rate constant and reaction rate for the Bahama Bank seawater was *k*_*D*_ = 2.64 × 10^11^ M^−1^ s^−1^ and 1.15 × 10^−10^ (Table 1). The Bahama Bank seawater has Ω values for calcite (Ω = 3.7), aragonite (Ω = 2.7) and vaterite (Ω = 1.0) that are similar to those in typical seawater (Table 1). Accordingly, seawater in the tropical region is not conducive to homogeneous nucleation of any of the polymorphs (i.e., nucleation formation rates <1; Table 1). Following the release of cytoplasmic ${\text{HCO}}_{3}^{-}$ from lysed cells, the nucleation activation energies for calcite (12-fold), aragonite (18-fold) and vaterite (2.6 × 10^4^-fold) decreased significantly and the Ω values increased to values (calcite: 90.7; aragonite: 65.5; vaterite: 25.2) similar to those calculated for typical seawater (Table 1). These post-lysis conditions were not favorable for the homogeneous nucleation of calcite or aragonite, but energetically favorable for the nucleation of vaterite at a rate of 1.03 × 10^26^ nuclei cm^−3^ s^−1^, with a critical nuclei size of 6 CaCO_3_ molecules, that takes ~2.0 × 10^−9^ s to occur (Table 1).

In the Makarov Basin, the seawater is much colder with a significantly lower pH among the two bodies of seawater. The second order reaction rate constant and reaction rate for the Makarov Basin was *k*_*D*_ = 2.94 × 10^11^ M^−1^ s^−1^ and 1.04 × 10^−10^ (Table 1). The native seawater in this region does not favor the nucleation of any of the CaCO_3_ polymorphs as all three were in an under saturated state (Table 1). The release of the cytoplasmic ${\text{HCO}}_{3}^{-}$ following viral lysis did elevate calcite (Ω = 15.1), aragonite (Ω = 10.3), and vaterite (Ω = 3.5) to supersaturated states. However, these levels of supersaturation were not sufficient to drive the nucleation of any of the polymorphs as all had nuclei formation rates <1 nuclei cm^−3^ s^−1^.

The microbially induced homogenous nucleation of the calcium carbonate proposed here contributes to our understanding of the earliest stage of a diagenetic process for carbonate sediments: nucleation. For example, in efforts to explain the occurrences of large carbonate precipitation events, like whitings, and their relationship to carbonate sediments microbial-based theories have been proposed that correlate these events with environmental stimuli such as phytoplankton blooms (Robbins and Blackwelder, 1992; Robbins et al., 1996; Thompson, 2000). Although a unifying mechanism of spontaneous precipitation has not been collectively or individually derived from these studies, a microbial surface precipitation model (i.e., heterogeneous nucleation) is commonly discussed. However, as previously noted, although this phenomenon is accepted as routinely occurring it would not alter the overall carbonate chemistry of seawater to the point where observable carbonate precipitation events occur (Morse et al., 1984, 2003), as previously noted.

Interestingly, all of these studies and discussions of calcium carbonate precipitation have one environmental aspect in common: relatively high abundances of photosynthesizing microbes during bloom events. Increases in cyanobacterial abundances in response to environmental stimuli have been shown to promote and enhance virally induced lytic events (Suttle and Chan, 1994; Danovaro et al., 2011). A global or regional scale virally induced release of the cyanobacteria-associated ${\text{HCO}}_{3}^{-}$ would not occur as a single event, episodic releases at the local-to-regional scale would occur in coastal zones where environmental stimuli (e.g., nutrient runoff, fresh water discharges, submarine groundwater discharges, etc.) promote rapid increases in cyanobacterial abundances. It is under these types of conditions that our proposed viral induced nucleation model would be a contributing process to the initiation and eventual production of carbonate sediments.

Lastly, the viral lysis model for calcium carbonate nucleation brings into consideration that the cytoplasm of photosynthesizing microorganisms is a repository for concentrated and reactive DIC, in the form of ${\text{HCO}}_{3}^{-}$, in seawater. The significance of this compartmentalized DIC becomes apparent when considered at global scales. For example, using an ocean surface area of 3.6 × 10^8^ km^2^, average photic zone depth of 150 m, average *Synechococcus* and *Prochlorococcus* abundance of 4.4 × 10^7^ cells L^−1^ (Worden et al., 2000) and the cell volumes and ${\text{HCO}}_{3}^{-}$ concentrations described previously, this population of cyanobacteria collectively retain ~1.0 GT of DIC on a global basis. This source of DIC is currently unaccounted for in carbon budgets.

## Author contributions

The authors have made substantial and equal, direct and intellectual contributions to the work, and approved it for publication.

### Conflict of interest statement

The authors declare that the research was conducted in the absence of any commercial or financial relationships that could be construed as a potential conflict of interest.
